# Zonal analysis in contemporary aesthetic orthognathic surgical planning

**DOI:** 10.1186/s40902-023-00379-5

**Published:** 2023-02-03

**Authors:** Mehmet Manisali, Farhad B. Naini

**Affiliations:** 1 Maxillofacial Unit, St George’s University Hospital, London, UK; 2 Kingston and St George’s University Hospitals, Galsworthy Road, KT2 7QB London, UK

**Keywords:** Orthognathic surgery, Three-dimensional, Virtual surgical planning

## Abstract

**Background:**

The purpose of this article is to introduce the concept of zonal analysis in orthognathic surgical planning.

**Case presentation:**

The importance of developing this concept of facial zonal analysis is because grouping together elements of aesthetic importance in a region in a systematic manner allows for accurate diagnosis and logical treatment planning. An orthognathic case presentation is described to demonstrate this concept.

**Discussion:**

The three facial zones described are related in terms of the presenting problem and in relation to the changes envisaged with each planned surgical movement. The importance of developing this concept of facial zonal analysis is because grouping together elements of aesthetic importance in a region in a systematic manner allows for accurate diagnosis and logical treatment planning. Consideration is given to the negative features that require improvement and the positive features that the clinician and patient would prefer not to alter. It also provides an organised tool for postoperative comparison of results. The analysis and synthesis of the information provided from this approach can aid contemporary orthognathic surgical planning.

## Background

The cardinal steps for training in aesthetic orthognathic surgical planning are described in Table 1. These are all laudable aims for any higher specialist trainee with an interest in orthognathic and craniofacial surgery. Every clinician should aim to improve the sense of aesthetic appreciation, with broad training in the facial aesthetic analysis [1], and the development of a general interest in art and artistic appreciation. It is often said that the appreciation of art is an art itself, i.e. something to be learnt and cultivated, and this is true for facial aesthetic appreciation in facial aesthetic and reconstructive surgery. In clinical practice, art is not just “for art’s sake”, but is for the betterment of the service we provide to our patients.

**Table 1:** Cardinal steps for training in aesthetic orthognathic surgical planning

Steps		
I	Envisage an aesthetic outcome	This is the most important step. The main ingredient required to do this is to *develop a sense of aesthetic appreciation*. This includes a thorough understanding of static and dynamic facial, smile and dental aesthetics
II	Develop dental-occlusal planning objectives accordingly	Dental-occlusal objectives should be developed according to the target facial aesthetic outcome. The key ingredient is good communication between the surgeon and the orthodontist. For example, should you fully or only partially decompensate the dentition prior to surgery?
III	Planning and execution of surgery	Treatment planning is predominantly based on clinical observation of the patient; this is true whether you go on to use conventional or 3D-VSP The effect of changing bony landmarks on soft tissues will always be variable and cannot be accurately predicted preoperatively Therefore, there is no substitute to knowledge and well-earned experience Virtual Surgical Planning (VSP) is extremely useful in experienced hands for the accurate planning and accurate technical execution and delivery of the planned outcome
IV	Critical appraisal of results	Without closing this loop, a clinician cannot progress in his or her work Clinical review of all cases should be mandatory Direct anthropometric analysis (e.g. alar base width, upper lip height etc.) is very useful for comparative purposes Postoperative 3D analysis Long-term follow-up of patients
V	Consideration of secondary procedures	Broad aesthetic training is highly beneficial, as secondary surgical procedures may be very useful following the primary orthognathic surgery

*3D-VSP* Three-dimensional virtual surgical planning

The emphasis in orthognathic planning is on the creation of a “composition” in the aesthetic and artistic sense of the word. The human face is comprised of different elements, referred to as facial units and subunits [1–3]. In art, the skill is in the artistic arrangement of the parts of a picture to create the most beautiful visual image, a process referred to as composition. In orthognathic practice, the clinician’s role is to plan such a composition in the dentofacial complex of each patient.

The purpose of this paper is to describe facial aesthetic analysis based on facial zones, which refers to the relevant adjacent regional subunits of the dentofacial complex. These facial zones are related in terms of the presenting problem, e.g. a retrusive maxilla may affect the upper lip and the lower part of the nose, and in relation to the changes envisaged with each planned surgical movement, e.g. a maxillary advancement will affect the shape and position of the upper lip and lower part of the nose. As such zonal analysis can be an important part of aesthetic planning in orthognathic and craniofacial surgery.

## Case presentation

### Factors influencing the composition

The concept of beauty is very difficult to define. It is not an absolute concept. It may change over time and may be influenced by gender, ethnicity and culture. From a clinical perspective, it is about harmony and balance [1]. The terms harmony and balance may be considered and envisaged, as described above, as the creation of a “composition”, i.e. understanding the structure and arrangement of the craniofacial complex, and the putting together of the comprising units and subunits of the craniofacial complex in order to create the best aesthetic and functional relationships. Any practitioner of aesthetics must undertake a lifetime challenge of analysing, understanding and creating such compositions. Unfortunately, although the quality of aesthetic appreciation is essential to achieving good outcomes, it is not emphasised or even necessarily recognised in higher specialist training. Therefore, it is each individual clinician’s responsibility to better themselves in this respect. Aesthetic appreciation should be part of a clinician’s education and may be learned, but requires time and effort.

It is beyond the scope of this paper to delve into each factor influencing decision making in orthognathic practice. However, briefly, it should be borne in mind that factors such as gender, ethnicity, age and culture all have an influence. For example, in terms of gender, a tall male patient with a preference for masculine facial features should not be made too feminine by excessive mandibular or chin set-back [4]. Stature, i.e. the combination of height and weight, is relevant, e.g. facial features and proportions should be in harmony with stature [5].

Other facial features, outside the areas to be altered by the proposed surgical procedures, should be analysed and considered. This is the basis of comprehensive craniofacial aesthetic analysis, which is beyond the scope of this paper [1–3], but is of paramount importance. Any region that is altered will have a relative influence on the aesthetic appearance of regions not altered with surgery.

Another prerequisite to achieving a successful aesthetic outcome is the ability to listen to and understand the patient concerns correctly. Accurately registering the patient’s concerns requires time and cannot be undertaken in a rushed consultation. Showing a patient photographs and videos, particularly of previously treated patients with similar presenting concerns (always used with the patient’s consent) is invaluable for this communication. The quest for improving our aesthetic sense is there to *process and guide* the patient’s aesthetic desires and expectations, not to override them.

What factors influence the aesthetic outcome in orthognathic surgery? These are the aesthetic components in facial zones — the term “components” in this respect does not refer to individual aesthetic subunits, but to facial contours, shapes, angles and relative relationships between adjacent facial regions. Facial proportions, facial symmetry, and dental aesthetics are extremely important but will not be covered in this paper; comprehensive explanations may be found elsewhere [1–3].

### Facial zones and zonal analysis

The importance of developing this concept of facial zonal analysis is because grouping together elements of aesthetic importance in a region in a systematic manner allows for accurate diagnosis and logical treatment planning. These should include the negative features that require improvement and, indeed, the positive features that the clinician and patient would prefer not to alter. The zones selected and described below correlate well with orthognathic surgical procedures. They also provide an organised tool for postoperative comparison of results.

**Fig. 1:**
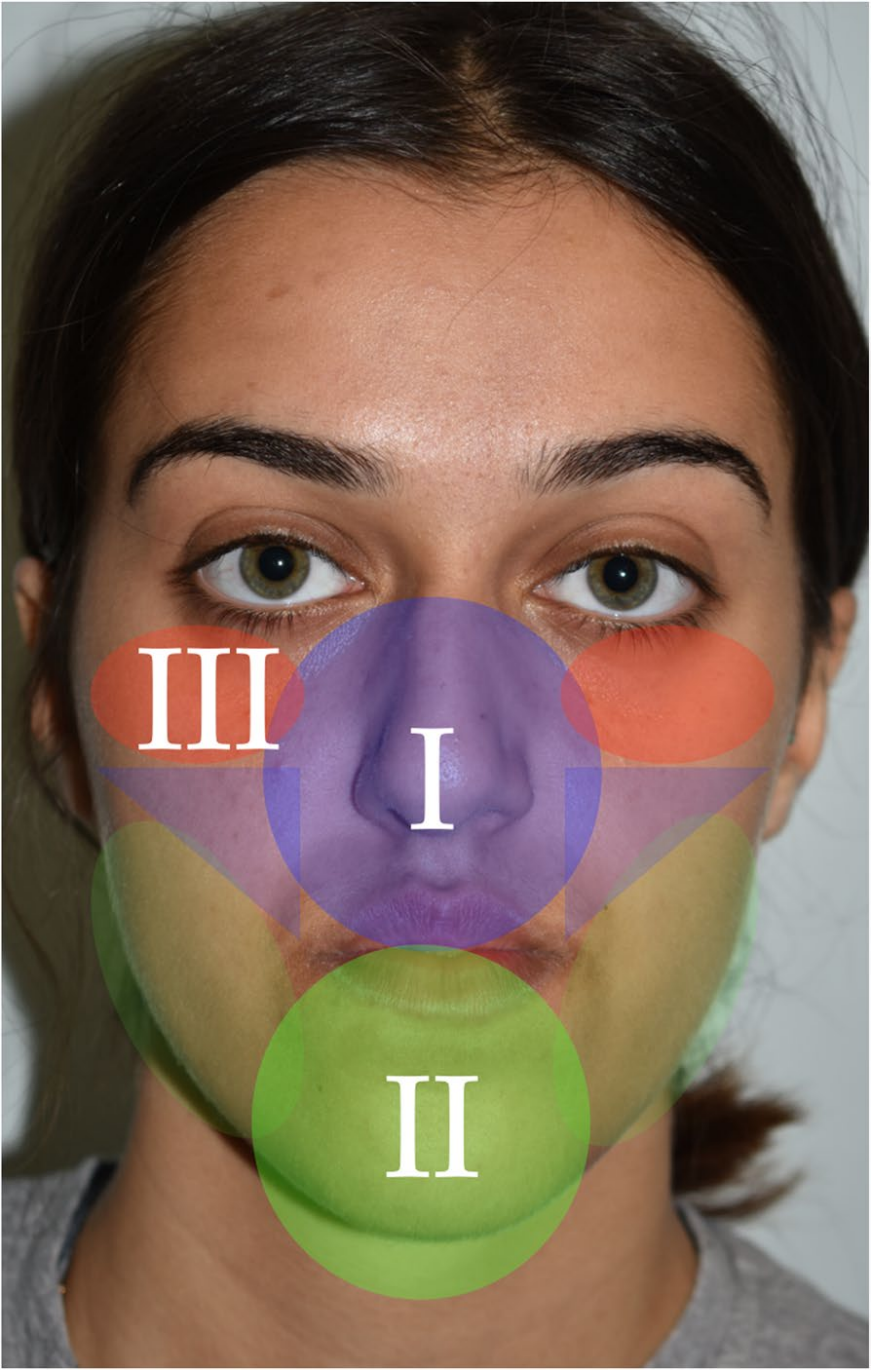
Facial zones used in zonal analysis. Zone I is the nasomaxillary zone (shown in the centre in purple), and its extended zones are the cheeks (shown as the bilateral triangles in purple). Zone II is the mandibular zone (shown in the centre in green), with extended zones bilaterally. Zone III is the malar zone

**Fig. 2:**
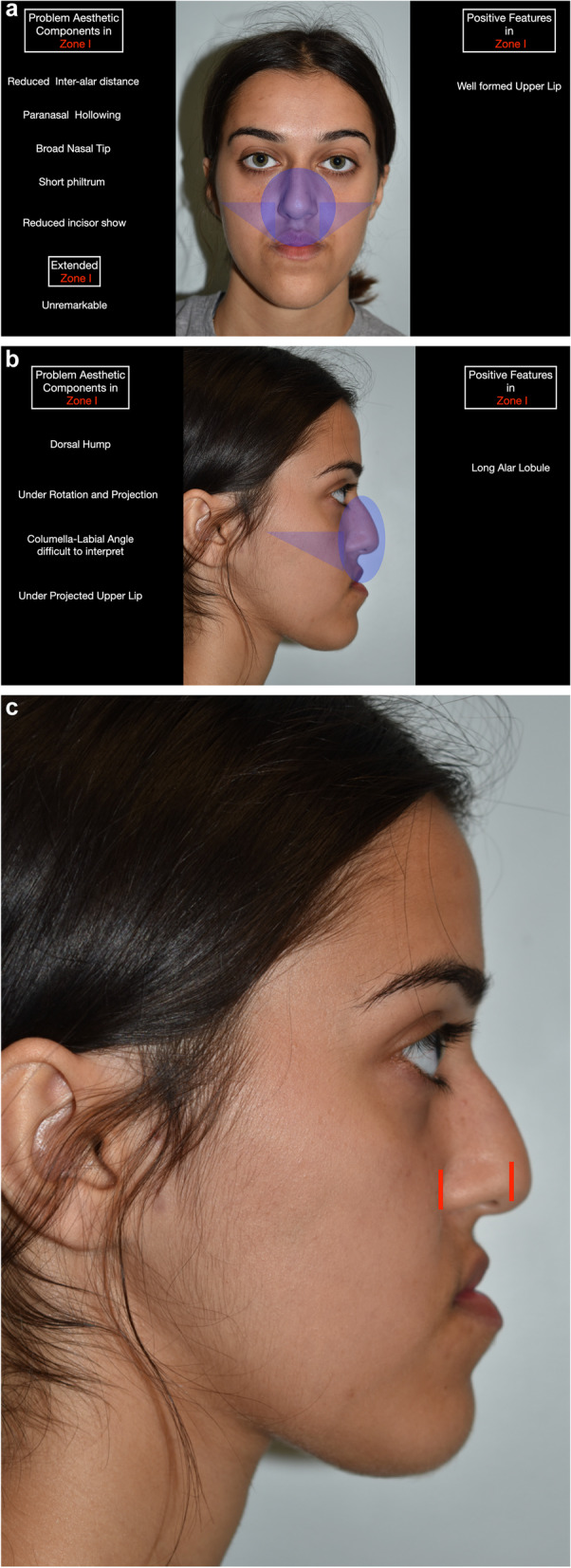
**a** Zone I (nasomaxillary zone), which includes the lower half of the nose, upper lip, paranasal regions and anterior maxillary dentition. The extended zones include the cheeks. **b** Zone I in profile view. **c** Profile view demonstrating the increased alar lobule length in zone I

**Fig. 3:**
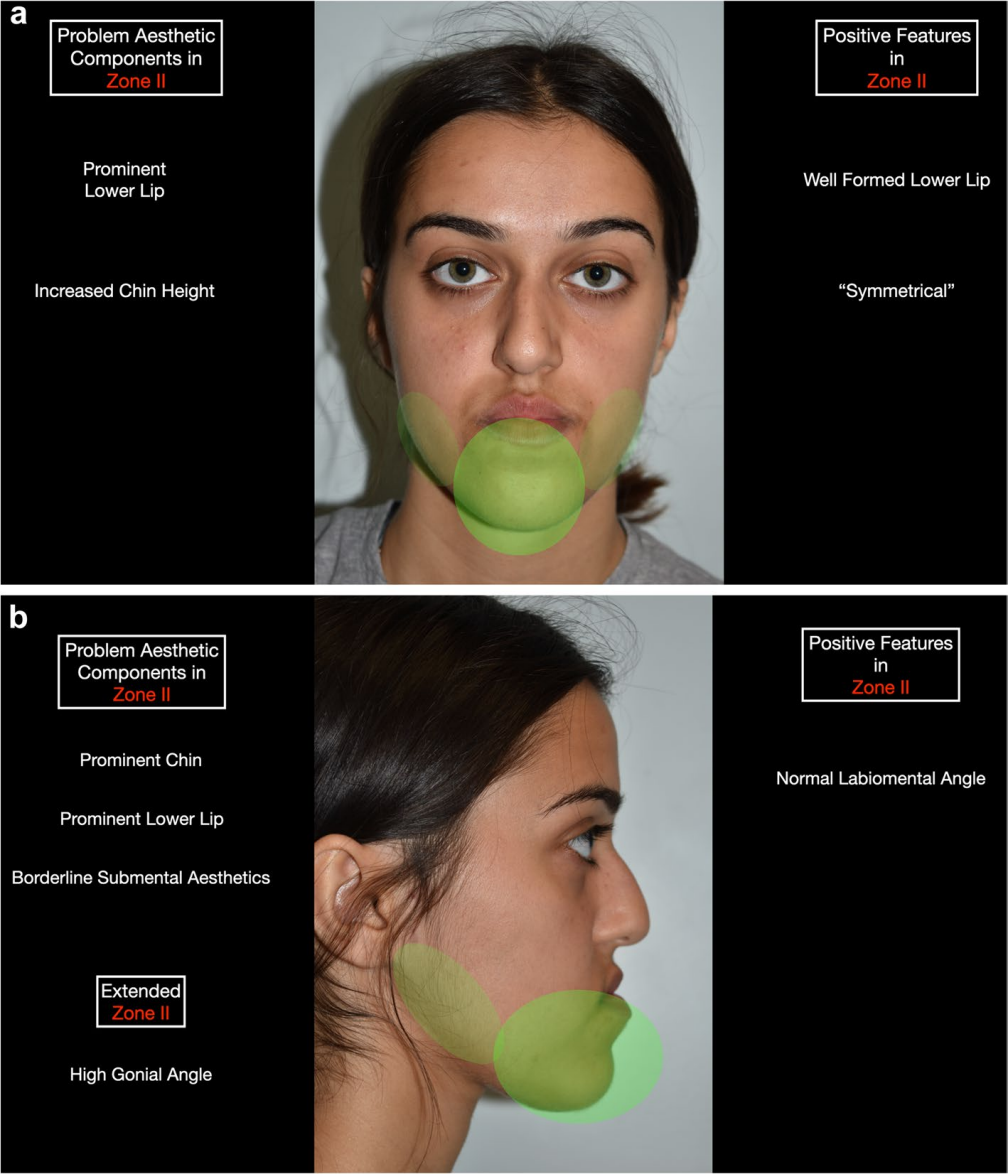
**a** Zone II (mandibular zone), which includes the anterior mandibular dentition, lower lip, labiomental fold and chin. The extended zones include the inferior borders of the mandible and gonial angles. **b** Zone II in profile view

**Fig. 4:**
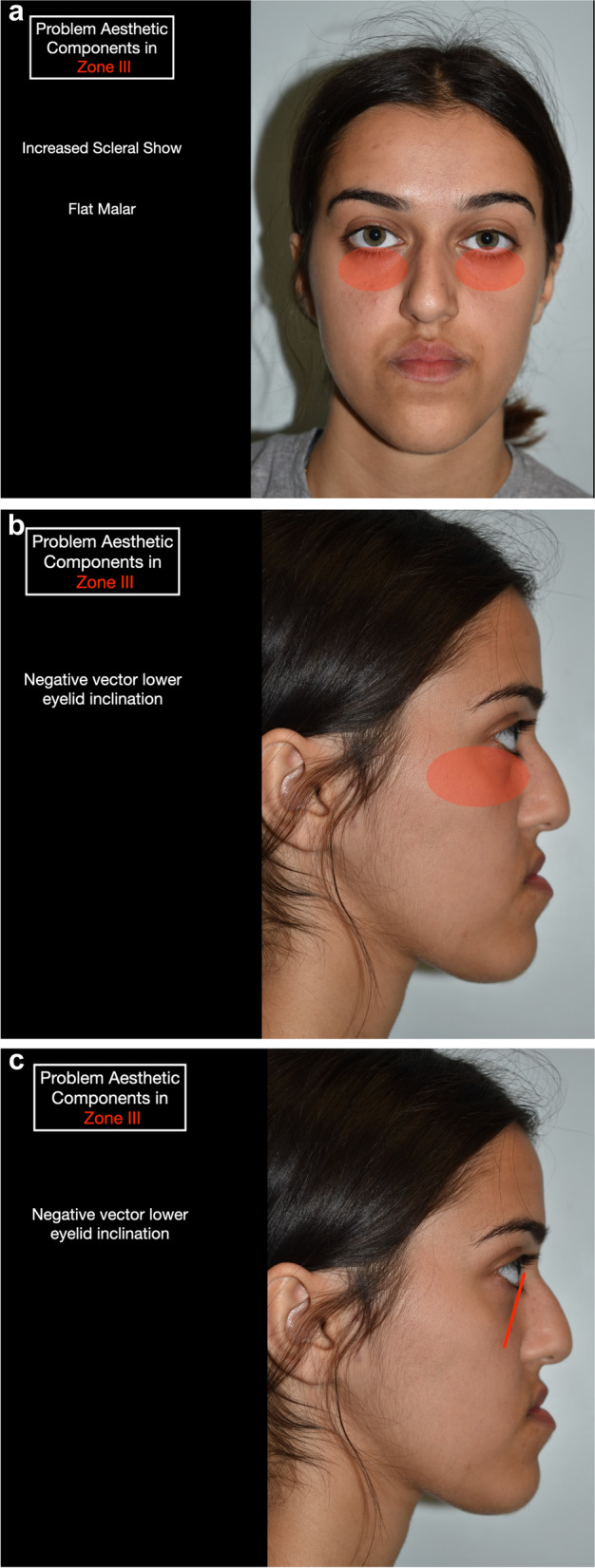
**a** Zone III (malar/zygomatic zone) in frontal view. **b** Zone III in profile view. **c** Negative vector of the lower eyelid evident in profile view in zone III

Zonal analysis is based on the following facial zones (Table 2) (Fig. 1), and the case described will be used as an example in this paper:

- Zone I (nasomaxillary zone) (Fig. 2): this includes the lower half of the nose, upper lip, paranasal regions and anterior maxillary dentition. The extended zones include the cheeks.
- Zone II (mandibular zone) (Fig. 3): this includes the anterior mandibular dentition, lower lip, labiomental fold and chin. The extended zones include the inferior borders of the mandible and gonial angles.
- Zone III (malar/zygomatic zone) (Fig. 4): this zone is somewhat in the periphery of conventional orthognathic surgery, but very important for diagnosis and planning, and in relation to higher level osteotomies. Although sometimes viewed as a somewhat supplementary area in conventional orthognathic surgery because the procedures do not directly affect the zygomatic region, nevertheless, indirect changes may occur in this region following Le Fort I osteotomies.

**Table 2:** Zonal analysis in orthognathic surgery (see Figs. 1, 2, 3, and 4)

	Principal aesthetic components in frontal view	Principal aesthetic components in profile view
Zone I (nasomaxillary)	• Nasal width • Paranasal hollowing • Upper lip height • Upper lip support and vermilion exposure • Incisor exposure – the foundation stone of treatment planning • Gingival exposure • Smile line • Buccal corridors • Dental midline • Maxillary occlusal plane cant	• Dorsal nasal profile • Nasal supratip morphology • Nasal rotation • Nasal projection • Alar lobule length • Columella-labial angle • Upper lip projection • Upper lip height • Lip balance (Fig. 5)
Extended Zone I	• Midfacial fat, e.g. the buccal fat pad • Midface soft tissue bunching – important for comparative purposes between pre and postoperative frontal views following maxillary impaction	
Zone II (mandibular)	• Lower incisor exposure • Lip support • Labiomental fold morphology • Chin width • Chin height • Chin symmetry • Mentalis activity and form	• Lip support • Labiomental fold morphology • Chin position • Chin morphology • Mentalis activity and form • Submental contour • Submental length • Submental-cervical angle • Lip balance
Extended Zone II	• Asymmetries in the inferior border and gonial angle regions • Masseteric hypertrophy or atrophy • Intergonial distance	Antegonial notch
Zone III (zygomatic)	• Flattening of the malar area • Inferior scleral exposure • Bizygomatic facial width	• Malar projection • Eye projection

**Fig. 5:**
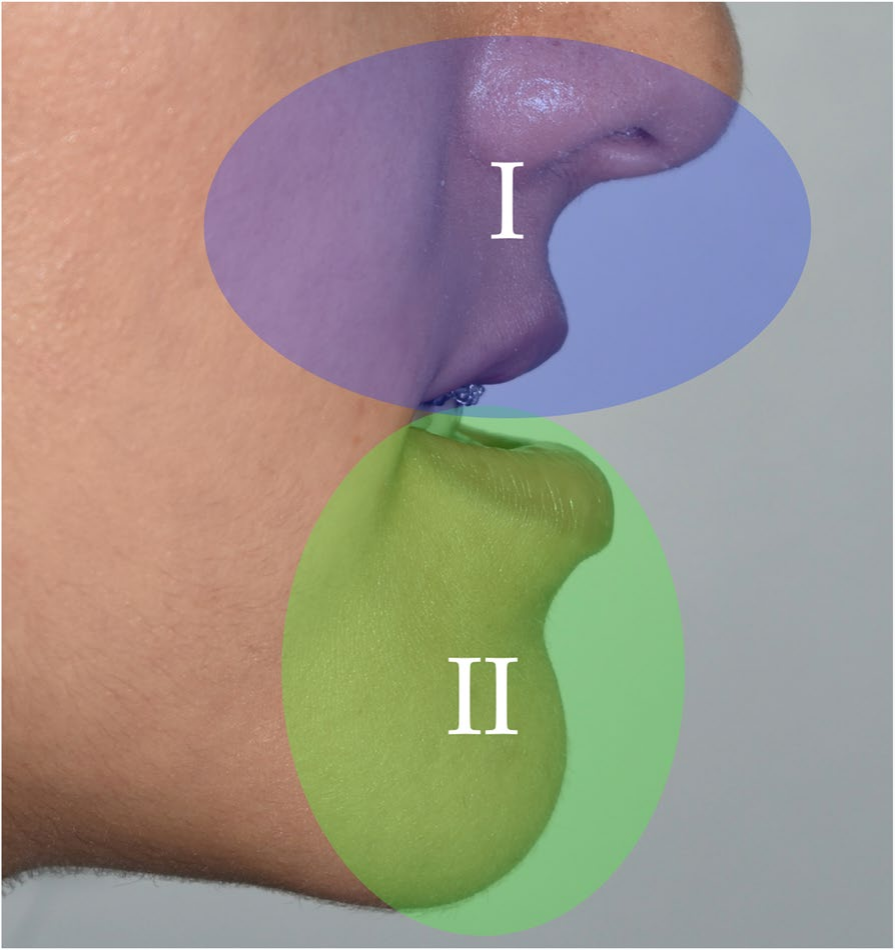
Lip balance is an important aesthetic component that falls between zones I and II

### Management of facial aesthetic components

Within each facial zone, the orthognathic team should select the components to change, select components to preserve, and warn the patient about the components that may worsen with each planned surgical procedure (Table 3).

**Table 3:** Impact of surgical procedures in different zones

	Primary procedures	Ancillary procedures	Secondary procedures	Potentially detrimental procedures^a^
Zone I	Le Fort I osteotomy High-level osteotomies Segmental osteotomies Distraction osteogenesis	ANS reduction Pyriform guttering	Rhinoplasty Fat grafting	Alar base cinch suture V–Y closure Excision of buccal fat pad
Zone II	Mandibular osteotomy (usually sagittal split ramus osteotomy) Osseous genioplasty Segmental osteotomies Distraction osteogenesis Rotational movements		Fat grafting Onlay implant Facelift Neck lift Liposuction	
Zone III	High-level osteotomies		Fat grafting Inferior orbital rim implant Malar implant Midface lift	

*ANS* Anterior nasal spine

^a^This is based on clinical experience and views about the potential detrimental nature of such procedures may vary (see text for further explanation)

It is important to stress that the three procedures listed in Table 3 as potentially detrimental procedures are all used frequently by many orthognathic surgeons. Therefore, it is necessary to specify why we feel that these procedures can be potentially detrimental. The alar base cinch suture, if used indiscriminately, can lead to unwanted nasal tip rotation, can create conversion in alar orientation, and may have long-term stability issues. The V–Y lip closure may lead to over protrusion of the central part of the upper lip, and may also have long-term stability issues. Buccal fat pad excision may create hollowing in an uncontrolled location, lead to loss of volume ahead of facial ageing, and can lead to asymmetry.

It is also important to make a list of the ‘precious’ aesthetic components, which are the most important aesthetic parameters. These include the following:

- Lip balance – this describes the aesthetic relationship between the upper and lower lips in frontal and profile view.
- Lip-incisor relationship – this describes the relationship between the maxillary incisors and the upper lip, particularly the incisor exposure in repose.
- Nasal changes – In particular, this includes changes to the nasal alar base width in the frontal view and changes to the nasal tip position in the profile view.
- Columella-labial angle – changes to the inclination and sagittal position of the upper lip occur following maxillary osteotomies, and changes occur to the nasal columella position, causing an overall change to this angle.
- Labiomental fold contour – the transition between the lower lip, through the labiomental fold region and to the soft tissue chin is one of the most important aesthetic parameters in zone II, which can change depending on the inclination and position of the mandibular incisors and changes to the sagittal and/or vertical position of the chin or lower anterior face height.
- Submental contour – this region can change with the sagittal movement of the mandible and/or chin.

The clinician should keep all these parameters in mind when planning orthognathic procedures. Ideally, the aesthetics of these regions should improve, and as far as possible, should not deteriorate. Ancillary procedures may be undertaken during the surgery, e.g. anterior nasal spine reduction or pyriform guttering. When potential negative results may occur in any of these regions, the patient should be informed and prepared prior to surgery, and discussion of potential secondary procedures undertaken.

### Likely areas of error in aesthetic planning

There are a number of areas of potential error in orthognathic surgical treatment planning. Some of the most important to avoid are the following:

- Excessive or reduced maxillary incisor exposure – this usually results from poorly planned vertical or sagittal positioning of the maxilla.
- Deviated septum – this is usually due to a surgical error in not trimming the inferior part of the nasal septum prior to maxillary superior repositioning at the Le Fort I level.
- Overcorrection in the sagittal direction – excessive maxillary advancement (usually anything over 5–6 mm) will have detrimental aesthetic effects.
- Poor submental contour – this is usually due to excessive mandibular or chin set-back, leading to bunching of the submental soft tissues and potentially a double-chin type appearance.
- ‘Operated look’ in the midface – usually creating a “bunched up” appearance of the midfacial soft tissues.
- Over-shortening of the midface – this is due to excessive maxillary impaction at the Le Fort I level.
- Poor management of facial height – facial height proportions should be planned and managed carefully. Sagittal and vertical skeletal repositioning will affect the mid and lower facial heights.
- Poor management of asymmetries – maxillary, mandibular and chin asymmetries are notoriously difficult to assess and manage. 3D-VSP can be a very useful additional tool when planning the correction of asymmetries [6].

### The process of aesthetic planning in practice

**Fig. 6:**
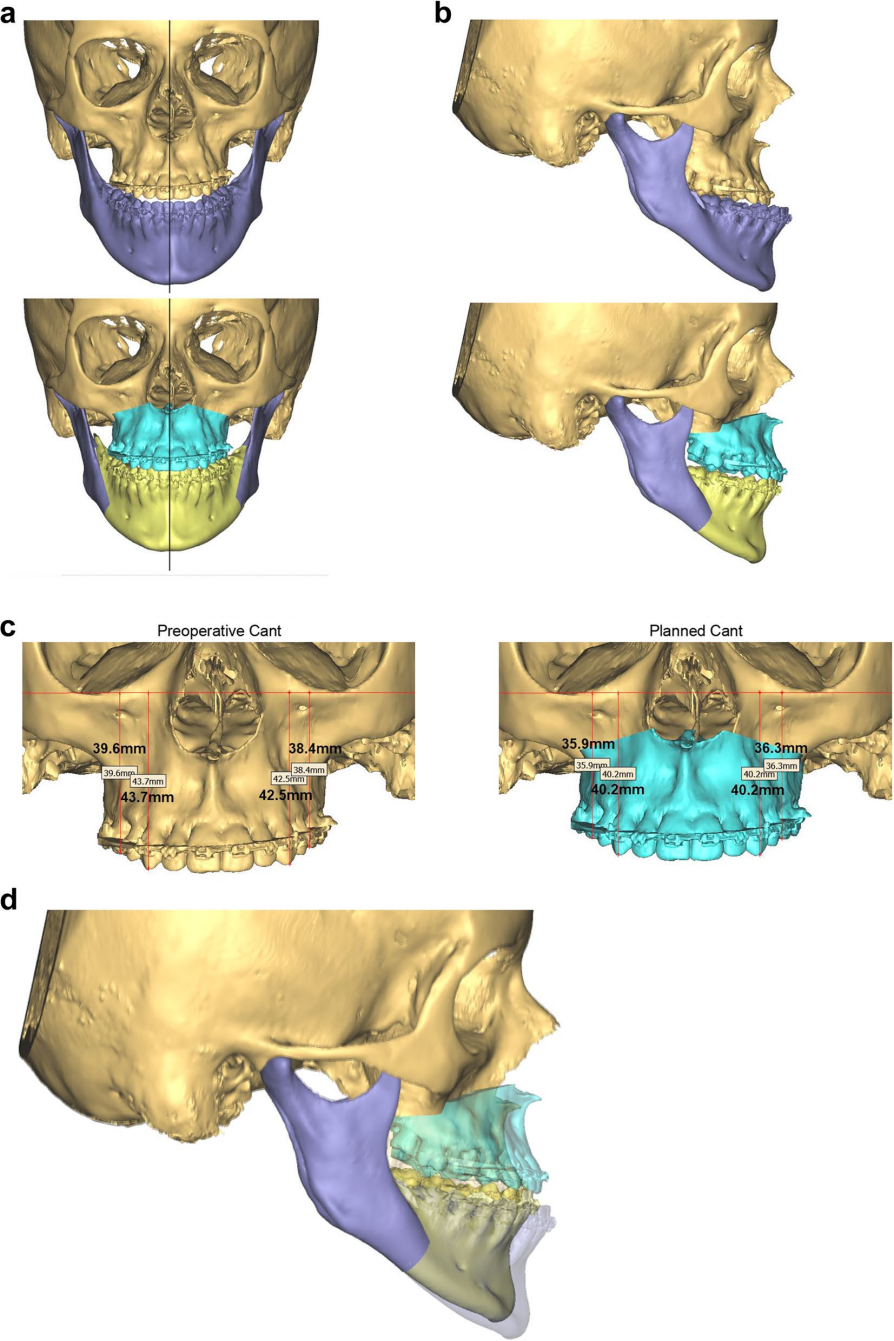
**a** 3D-VSP used to confirm the treatment plan, requiring bimaxillary surgery, with maxillary advancement and transverse occlusal plane cant correction and mandibular set-back to Class I skeletal and Class I incisor relationship. Preoperative position shown above; planned position shown below. **b** Profile planning. Preoperative position shown above; planned position shown below. **c** Transverse maxillary occlusal plane cant correction planning. **d** Superimposition of the preoperative and planned skeletal positions

Constructing the treatment plan requires a number of parameters to be evaluated carefully. These are assessed clinically and re-evaluated during the 3D-VSP meeting (Fig. 6):

- Determination of the ideal maxillary position
- Imaginary adaptation of the mandible to the new maxillary position
- Adaptation of the orthodontic plan according to the planned skeletal position (i.e. according to the above)
- Evaluation of the chin
- Evaluation of the nose
- Evaluation of the malar and other areas

The ideal maxillary position is determined by evaluation of the following parameters:

- Desired lip-incisor relationship
- Appropriate support of the upper lip
- Correction of paranasal deficiency
- Contribution to nasal aesthetics (or damage limitation)
- Management of the transverse dimension

The ideal mandibular position is dictated by the predetermined maxillary position, but should be made with carefully consideration of the following parameters:

- Appropriate support of the lower lip
- Desired labiomental fold contour
- Review of the chin position
- Proportionality of the facial heights
- Improved (or preserved) submental aesthetics

While dealing with the above parameters, it is potentially possible to have missed certain important parameters. Therefore, a checklist is important, which asks the following questions:

- Is there an anterior open bite tendency?
- Is there a transverse problem?
- Is there an occlusal plane cant?
- What is the status of the dental and facial midlines?
- Does the chin require modification?
- Is the detailed treatment plan appropriate for the age, sex and ethnic background of the patient?
- Could there be anticipated functional problems (OSA, TMJ)?
- Have you informed the patient about the potential requirement for secondary procedures?

It is important to acknowledge that the first five parameters listed above may be assessed very accurately during the 3D-VSP planning session, which is one of the most significant improvements when using 3D-VSP compared with conventional planning.

There are a number of final general questions that should be considered:

- Have we managed to avoid excessive skeletal moves?
- Have we managed to reduce the treatment plan to its simplest form?
- Would single-jaw surgery be an alternative?

**Fig. 7:**
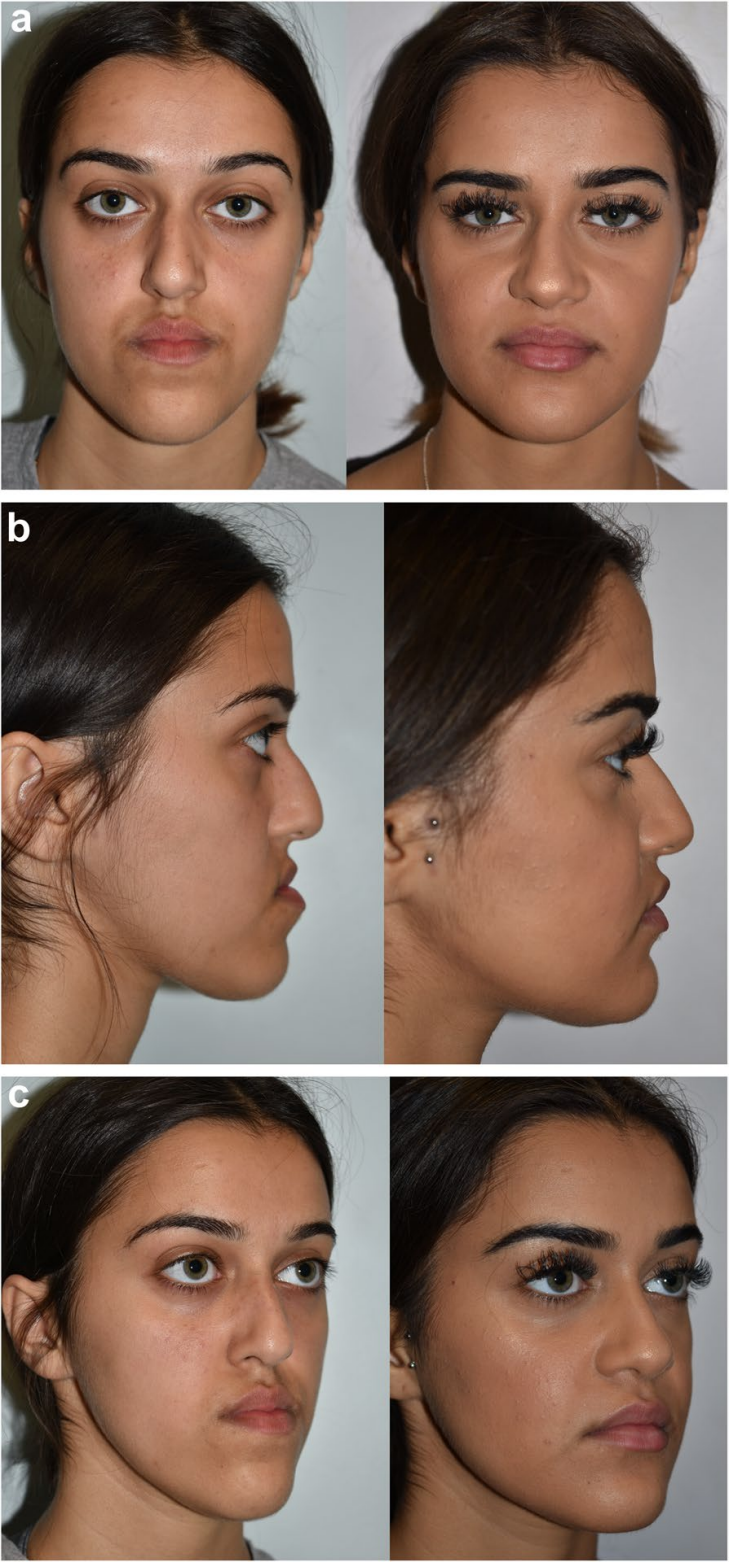
**a** Preoperative and end of treatment frontal images. **b** Preoperative and end of treatment profile images. **c** Preoperative and end of treatment oblique lateral images

Once all these questions have been asked, reflected upon, and satisfactorily answered, the plan is finalised (Fig. 7). For those undertaking conventional orthognathic surgery, the planning process moves to the maxillofacial technologist for model surgery and surgical splint fabrication. If undertaking 3D-VSP, surgical stents are prepared for surgery.

## Discussion

The orthognathic team should develop the ability to picture a ‘composition’, but remember that the composition is not an absolute but is variable. The following points merit consideration for every orthognathic patient:

- Understand the patient’s concerns and respect them.
- Planning is as much an exercise of not creating new problems as it is of solving the presenting issues.
- Avoid big moves (everything should be in moderation).
- Embrace the concept of incomplete incisor decompensation when indicated. One of the key questions at the start of treatment is what will be the anticipated incisor overjet or reverse overjet at the end of the presurgical orthodontic preparation phase of treatment? Accurate planning will avoid the requirement for excessive skeletal movements.
- Remember that the soft tissue envelope has its limits.
- For a natural looking result, the skeleton should be sitting comfortably in the soft tissue envelope.
- Slight skeletal under correction is often better than over correction from a facial aesthetic point of view.
- Inform patients about the potential need for secondary procedures at the start of treatment and during the consent process.
- A broad repertoire of facial aesthetic procedures is a major advantage in orthognathic surgery practice.

The concept of zonal analysis in orthognathic surgery is useful as it allows the systematic grouping together of parameters of aesthetic importance in each region of the face. This allows accurate diagnosis and logical treatment planning. The technique considers the negative dentofacial features that require improvement and the positive features that the clinician and patient would prefer to maintain. These zones correlate well with orthognathic surgical procedures and provide an organised tool for postoperative comparison of results.

## Data Availability

Not applicable.

## Abbreviations

3D: Three-dimensional
OSA: Obstructive sleep apnoea
TMJ: Temporomandibular joint
VSP: Virtual surgical planning

## References

[1] Naini FB (2011). Facial Aesthetics: Concepts and Clinical Diagnosis.

[2] Naini FB, Gill DS, Naini FB, Gill DS (2017). Patient evaluation and clinical diagnosis. Orthognathic Surgery: Principles, Planning and Practice.

[3] Naini FB, Gill DS, Naini FB, Gill DS (2017). Principles of orthognathic treatment planning. Orthognathic Surgery: Principles, Planning and Practice.

[4] Gill DS, Lloyd T, East C, Naini FB (2017). The facial soft tissue effects of orthognathic surgery. Facial Plast Surg.

[5] Naini FB, Cobourne MT, McDonald F, Donaldson AN (2008). The influence of craniofacial to standing height proportion on perceived attractiveness. Int J Oral Maxillofac Surg.

[6] Donaldson CD, Manisali M, Naini FB (2021). 3D virtual surgical planning (3D-VSP) in orthognathic surgery: Advantages, disadvantages and pitfalls. J Orthod.

